# Waller and De Watteville on the Electrotonus of Human Nerves

**Published:** 1882-07

**Authors:** 


### WALLER AND DE WATTEVILLE ON THE ELECTROTONUS OF HUMAN NERVES.

The results obtained by these observers, after a long and patient investigation, are embodied in a memoir, communicated to the Royal Society by Prof. Burdon Sanderson, and of which an abstract was read at the last meeting. Many experiments have been made in Germany during the last fifteen years, with a view to demonstrate on living human nerve the phenomena so well known since Pflüger's classical researches. Owing to imperfect methods, however, the results had been as scanty as conflicting. Drs. Waller and De Watteville have succeeded, however, in demonstrating most clearly that the same alterations of irritability which are observed in the exsected frog's nerve, both during and after the passage of a galvanic current, occur in the living nerve. Some of these alterations seem indeed to be far more marked in the latter than in the former ; and the perfected methods they employed gave remarkably clear and uniform results. They tested the irritability of the polarized nerves, not only by means of galvanic and faradaic stimuli, but mechanical stimuli, also a novel and important feature in their experiments. The consideration of the numerous sources of fallacy to which experiments on the human body are exposed led them to investigate a number of collateral phenomena of interest. Whether any immediate application of their results to electro-diagnosis and therapeutics is possible remains to be seen, but in the meanwhile we are glad to observe that their memoir is the first contribution ever made in England to the cause of scientific electro-therapeutics.—*Med. Press and Circular.*

